# Supplementary material for: Task-specific odorant receptor expression in worker antennae indicates that sensory filters regulate division of labor in ants
Source: Commun Biol. 2023 Oct 2;6:1004. doi: 10.1038/s42003-023-05273-4 (PMC10545721; doi:10.1038/s42003-023-05273-4)
Supplement: Supplementary file 1 — Supporting Information [file 42003_2023_5273_MOESM1_ESM.pdf]

Table S1. List of candidate genes related to behavior phenotypes.

Gene Annotation	Locus (*T. longispinosus*)	Organism reference	Locus reference	Orthogroup	Task	Tissue	Pathways and genes	References
protein takeout isoform	DBV15_08771	*Camponotus floridanus*	LOC105257386	OG0007297	Nurse	Brain	JH: signalling	[1]
vitellogenin-1 (Vg conventional)	LOC112466671	*Harpegnathos saltator*	LOC105181726	OG0003549	Nurse	Brain	Vitellogenin	[1–4]
vitellogenin-2-like (Vg like-A)	DBV15_03138	*H. saltator*	LOC105191299	OG0009030	Nurse	Brain	Vitellogenin	[2,3]
farnesyl pyrophosphate synthase	LOC112466955	*Drosophila melanogaster*	Fpps	OG0000401	Nurse	Brain	JH: biosynthesis	[5]
venom carboxylesterase-6 (Jhe1)	DBV15_11528	*H. saltator*	LOC112590013	OG0002262	Nurse	Brain/Antenna	JH: regulation of JH biosynthesis	[1,3]
ribosomal protein S6	DBV15_00490	*D. melanogaster*	RpS6	OG0007546	Nurse	Brain/Antenna	IIS / TOR	[5]
dopamine 1-like receptor 2	DBV15_07611	*D. melanogaster*	Dop1R2	OG0001377	Nurse	Antenna	bioagenic amines	[6]
octopamine beta2 receptor	DBV15_10418	*D. melanogaster*	Octbeta2R	OG0000466	Nurse	Antenna	bioagenic amines	[6]
aldehyde dehydrogenase	DBV15_01763	*D. melanogaster*	Aldh-III	OG0000175	Nurse	Antenna	JH: biosynthesis	[5]

cyclin G	DBV15_03888	*D. melanogaster*	CycG	OG0003395	Nurse	Antenna	IIS / TOR	[5]
happyhour	DBV15_11653	*D. melanogaster*	hppy	OG0000352	Nurse	Antenna	IIS / TOR	[5]
insulin receptor isoform (InRL)	DBV15_10224	*H. saltator*	LOC105183944	OG0001487	Nurse	Antenna	IIS	[3]
Pi3K21B	DBV15_03081	*D. melanogaster*	Pi3K21B	OG0002238	Nurse	Antenna	IIS	[5]
Ras oncogene at 64B	DBV15_02802	*D. melanogaster*	Ras64B	OG0007075	Nurse	Antenna	IIS / TOR	[5]
sarcoplasmic calcium-binding protein 2	DBV15_05072	*D. melanogaster*	Scp2	OG0005773	Nurse	Antenna	JH: metabolism	[5]
shaggy	DBV15_09601	*D. melanogaster*	sgg	OG0000404	Nurse	Antenna	IIS / TOR	[3,5]
allatostatin A	LOC112454443	*D. melanogaster*	AstA	OG0007104	Forager	Brain	JH: regulation of JH biosynthesis	[5]
insulin-like growth factor I (IGF1)	LOC112454447	*H. saltator*	LOC105186969	OG0004678	Forager	Brain/Antenna	IIS	[3,4]
5-hydroxytryptamine (serotonin) receptor 2A	DBV15_11483	*D. melanogaster*	5-HT2A	OG0000533	Forager	Antenna	bioagenic amines	[6]
octopamine receptor in mushroom bodies	LOC112465659	*D. melanogaster*	Oamb	OG0001501	Forager	Antenna	bioagenic amines	[6]
tyramine beta hydroxylase	DBV15_00422	*D. melanogaster*	Tbh	OG0006646	Forager	Antenna	bioagenic amines	[6]

density regulated protein	DBV15_10283	*D. melanogaster*	DENR	OG0004331	Forager	Antenna	IIS / TOR	[5]
dreadlocks	DBV15_03530	*D. melanogaster*	dock	OG0001746	Forager	Antenna	IIS / TOR	[5]
juvenile hormone-inducible protein 26	DBV15_02220	*D. melanogaster*	JhI-26	OG0000832	Forager	Antenna	JH: regulation of JH biosynthesis	[5]
Krueppel homolog 1-like	DBV15_06330	*H. saltator*	LOC112589664	OG0008125	Forager	Antenna	JH: signalling	[3,4]
rapamycin-insensitive companion of Tor	DBV15_07973	*D. melanogaster*	rictor	OG0003860	Forager	Antenna	IIS / TOR	[5]
SAPK-interacting protein 1	DBV15_07343	*D. melanogaster*	Sin1	OG0005622	Forager	Antenna	IIS / TOR	[5]

References

1. Das B, de Bekker C. Time-course RNASeq of *Camponotus floridanus* forager and nurse ant brains indicate links between plasticity in the biological clock and behavioral division of labor. BMC Genomics. 2022;23(1):1–23.
2. Kohlmeier P, Feldmeyer B, Foitzik S. *Vitellogenin-like A*–associated shifts in social cue responsiveness regulate behavioral task specialization in an ant. PLoS Biol. 2018;16(6):1–26.
3. Gospocic J, Glastad KM, Sheng L, Shields EJ, Berger SL, Bonasio R. Kr-h1 maintains distinct caste-specific neurotranscriptomes in response to socially regulated hormones. Cell. 2021;184(23):5807-5823.e14.
4. Opachaloemphan C, Mancini G, Konstantinides N, Parikh A, Mlejnek J, Yan H, et al. Early behavioral and molecular events leading to caste switching in the ant *Harpegnathos*. Genes Dev. 2021;35(5–6):410–24.
5. Korb J, Meusemann K, Aumer D, Bernadou A, Elsner D, Feldmeyer B, et al. Comparative transcriptomic analysis of the mechanisms underpinning ageing and fecundity in social insects. Philos Trans R Soc B Biol Sci. 2021;376(1823).
6. Gramates L, Agapite J, Attrill H, Calvi B, Crosby M, dos Santos, et al. FlyBase: a guided tour of highlighted features. Genetics. 2022;220(4):iyac035.

Table S2. List of enriched GO biological process terms of the DEG in the brain and the antenna.

Gene Set (DEG)	Tissue	GO ID	Term	Annotated	Significant	Expected	Fisher (*p*-value)
Nurse	Brain	GO:0006412	translation	73	19	1.79	0.000
Nurse	Brain	GO:0006879	cellular iron ion homeostasis	5	2	0.12	0.006
Nurse	Brain	GO:0009056	catabolic process	155	6	3.79	0.024
Nurse	Brain	GO:0030155	regulation of cell adhesion	1	1	0.02	0.025
Nurse	Brain	GO:0010040	response to iron (II) ion	1	1	0.02	0.025
Nurse	Brain	GO:0030245	cellulose catabolic process	1	1	0.02	0.025
Nurse	Brain	GO:0009204	deoxyribonucleoside triphosphate catabolic process	1	1	0.02	0.025
Nurse	Brain	GO:0006559	L-phenylalanine catabolic process	2	1	0.05	0.048
Nurse	Brain	GO:0006572	tyrosine catabolic process	2	1	0.05	0.048
Nurse	Brain	GO:0007160	cell-matrix adhesion	2	1	0.05	0.048
Nurse	Brain	GO:0006546	glycine catabolic process	2	1	0.05	0.048
Nurse	Brain	GO:0019236	response to pheromone	2	1	0.05	0.048
Nurse	Brain	GO:0030334	regulation of cell migration	2	1	0.05	0.048
Forager	Brain	GO:0006518	peptide metabolic process	89	4	0.96	0.001

Forager	Brain	GO:0006431	methionyl-tRNA aminoacylation	1	1	0.01	0.011
Forager	Brain	GO:0032481	positive regulation of type I interferon production	1	1	0.01	0.011
Forager	Brain	GO:0045454	cell redox homeostasis	2	1	0.02	0.022
Forager	Brain	GO:0046855	inositol phosphate dephosphorylation	4	1	0.04	0.043
Nurse	Antenna	GO:0006412	translation	73	22	8.93	0.000
Nurse	Antenna	GO:0006897	endocytosis	15	6	1.83	0.006
Nurse	Antenna	GO:0006006	glucose metabolic process	10	4	1.22	0.006
Nurse	Antenna	GO:0051726	regulation of cell cycle	9	3	1.1	0.015
Nurse	Antenna	GO:0015012	heparan sulfate proteoglycan biosynthetic process	3	2	0.37	0.040
Forager	Antenna	GO:0046854	phosphatidylinositol phosphorylation	6	4	0.57	0.001
Forager	Antenna	GO:0046855	inositol phosphate dephosphorylation	4	3	0.38	0.003
Forager	Antenna	GO:0005975	carbohydrate metabolic process	81	17	7.74	0.004
Forager	Antenna	GO:0045087	innate immune response	2	2	0.19	0.009
Forager	Antenna	GO:0009166	nucleotide catabolic process	4	3	0.38	0.025
Forager	Antenna	GO:0007229	integrin-mediated signaling pathway	9	3	0.86	0.045
Forager	Antenna	GO:0006166	purine ribonucleoside salvage	4	2	0.38	0.047

Table S3. Behaviors and positions annotations during nest scans. Nursing behavior highlighted in red was defined as the number of observations a worker antennating, grooming, feeding or carrying a brood item, and its position inside the nest (on the brood or near to the brood pile). Foraging behavior highlighted in blue was defined as the number of observations an individual was found outside the nest, collecting food or water.

**Position**	
Inside the nest	On the brood pile
	Near the brood pile
	Surroundings of the nest
	Near the entrance
Outside the nest	Chamber 1
	Chamber 2
	Chamber 3
**Behavior**	
Brood care	Antennation, grooming, feeding or carrying the brood
Nestmate care	Antennation, grooming, feeding or carrying a nestmate
Forager	Collecting food/drinking water outside the nest
Others	Resting
	Walking
	Being groomed or fed
	Grooming itself
