# Supplementary material for: Task-specific odorant receptor expression in worker antennae indicates that sensory filters regulate division of labor in ants
Source: Commun Biol. 2023 Oct 2;6:1004. doi: 10.1038/s42003-023-05273-4 (PMC10545721; doi:10.1038/s42003-023-05273-4)
Supplement: Supplementary file 2 — Description of Additional Supplementary Data [file 42003_2023_5273_MOESM2_ESM.docx]

**Description of Additional Supplementary Files**

**File name:** Supplementary Data 1

**Description:** Behaviors and positions annotations during nest scans.

**File name:** Supplementary Data 2

**Description:** Gene list with caste-differential expression, NCBI BlastX results, and gene read count of 28 RNA-seq samples.

**File name:** Supplementary Data 3

**Description:** OR subfamilies and orthogroups from the DEG lists between nurses and foragers in the antenna.

**File name:** Supplementary Data 4

**Description:** Summary of ant brain and antenna transcriptome data sets and mapping results.

**File name:** Supplementary Data 5

**Description:** List of orthogroups from the DEG list between nurses and foragers in Temnothorax longispinosus.
