# Supplementary material for: Task-specific odorant receptor expression in worker antennae indicates that sensory filters regulate division of labor in ants
Source: Commun Biol. 2023 Oct 2;6:1004. doi: 10.1038/s42003-023-05273-4 (PMC10545721; doi:10.1038/s42003-023-05273-4)
Supplement: Supplementary file 8 — Reporting Summary [file 42003_2023_5273_MOESM8_ESM.pdf]

nature portfolio

Corresponding author(s): Marcel A. Caminer

Last updated by author(s): 2023/08/03

# Reporting Summary

Nature Portfolio wishes to improve the reproducibility of the work that we publish. This form provides structure for consistency and transparency in reporting. For further information on Nature Portfolio policies, see our Editorial Policies and the Editorial Policy Checklist.

## Statistics

For all statistical analyses, confirm that the following items are present in the figure legend, table legend, main text, or Methods section.

| n/a | Confirmed | |
|---|---|---|
| ☐ | ☒ | The exact sample size (*n*) for each experimental group/condition, given as a discrete number and unit of measurement |
| ☐ | ☒ | A statement on whether measurements were taken from distinct samples or whether the same sample was measured repeatedly |
| ☒ | ☐ | The statistical test(s) used AND whether they are one- or two-sided<br>*Only common tests should be described solely by name; describe more complex techniques in the Methods section.* |
| ☒ | ☐ | A description of all covariates tested |
| ☒ | ☐ | A description of any assumptions or corrections, such as tests of normality and adjustment for multiple comparisons |
| ☒ | ☐ | A full description of the statistical parameters including central tendency (e.g. means) or other basic estimates (e.g. regression coefficient) AND variation (e.g. standard deviation) or associated estimates of uncertainty (e.g. confidence intervals) |
| ☒ | ☐ | For null hypothesis testing, the test statistic (e.g. *F*, *t*, *r*) with confidence intervals, effect sizes, degrees of freedom and *P* value noted<br>*Give P values as exact values whenever suitable.* |
| ☒ | ☐ | For Bayesian analysis, information on the choice of priors and Markov chain Monte Carlo settings |
| ☒ | ☐ | For hierarchical and complex designs, identification of the appropriate level for tests and full reporting of outcomes |
| ☒ | ☐ | Estimates of effect sizes (e.g. Cohen's *d*, Pearson's *r*), indicating how they were calculated |

*Our web collection on statistics for biologists contains articles on many of the points above.*

## Software and code

Policy information about availability of computer code

| | |
|---|---|
| Data collection | NA |
| Data analysis | NA |

For manuscripts utilizing custom algorithms or software that are central to the research but not yet described in published literature, software must be made available to editors and reviewers. We strongly encourage code deposition in a community repository (e.g. GitHub). See the Nature Portfolio guidelines for submitting code & software for further information.

## Data

Policy information about availability of data

All manuscripts must include a data availability statement. This statement should provide the following information, where applicable:

- Accession codes, unique identifiers, or web links for publicly available datasets
- A description of any restrictions on data availability
- For clinical datasets or third party data, please ensure that the statement adheres to our policy

RNAseq data have been deposited in NCBI with the BioProject accession codes PRJNA926589 (http://www.ncbi.nlm.nih.gov/bioproject/926589)

## Research involving human participants, their data, or biological material

Policy information about studies with human participants or human data. See also policy information about sex, gender (identity/presentation), and sexual orientation and race, ethnicity and racism.

| | |
|---|---|
| Reporting on sex and gender | NA |
| Reporting on race, ethnicity, or other socially relevant groupings | NA |
| Population characteristics | NA |
| Recruitment | NA |
| Ethics oversight | NA |

Note that full information on the approval of the study protocol must also be provided in the manuscript.

# Field-specific reporting

Please select the one below that is the best fit for your research. If you are not sure, read the appropriate sections before making your selection.

☐ Life sciences ☐ Behavioural & social sciences ☒ Ecological, evolutionary & environmental sciences

For a reference copy of the document with all sections, see nature.com/documents/nr-reporting-summary-flat.pdf

# Ecological, evolutionary & environmental sciences study design

All studies must disclose on these points even when the disclosure is negative.

| | |
|---|---|
| Study description | We test the novel hypothesis that social insect workers perform different tasks because they differ in their ability to detect task-related stimuli. We performed RNA-seq in the ant Temnothorax longispinosus to investigate gene expression differences between distinct behavioral phenotypes in the brain and in the antennae. We pooled seven individuas of the same behavior phenotype for each colony (number of colonies = 7) |
| Research sample | A total of seven colonies of the ant Temnothorax longispinosus were selected with an average colony size of 110 ± 31.5 workers |
| Sampling strategy | A total of 69 workers inside and 76 workers outside the nest were marked. We performed behavioral observations for five days |
| Data collection | June of 2018 |
| Timing and spatial scale | The collection behavior was taken in a period of time of 3-4 months (mid-March to late June) |
| Data exclusions | no data were excluded |
| Reproducibility | Seven replicates per treatment |
| Randomization | Individuals were randomized during dissections, RNA extractions and sequencing |
| Blinding | Blinding was not possible because we had to observe behavior to discriminate between forager and nurses |

Did the study involve field work? ☒ Yes ☐ No

## Field work, collection and transport

| | |
|---|---|
| Field conditions | We housed each colony in a plaster-floored nesting box divided into three chambers containing a single slide nest, in which the colony relocated. A slide nest is an artificial nesting site comprised of a small Plexiglas cavity sandwiched between two glass microscope slides |
| Location | Colonies were established at Johannes Gutenberg University in Mainz, under a 14 h:10 h light:dark photoperiod at 18°C to a 22°C E. N. Huyck |
| Access & import/export | Preserve, Rensselearville provided the collection permit. Import and export licences are not required |

| | |
|---|---|
| Disturbance | These ants live in small places like acorns and holes in sticks. Therefore the disturbances were minimal |

# Reporting for specific materials, systems and methods

We require information from authors about some types of materials, experimental systems and methods used in many studies. Here, indicate whether each material, system or method listed is relevant to your study. If you are not sure if a list item applies to your research, read the appropriate section before selecting a response.

## Materials & experimental systems

| n/a | Involved in the study | |
|---|---|---|
| ☒ | ☐ | Antibodies |
| ☒ | ☐ | Eukaryotic cell lines |
| ☒ | ☐ | Palaeontology and archaeology |
| ☐ | ☒ | Animals and other organisms |
| ☒ | ☐ | Clinical data |
| ☒ | ☐ | Dual use research of concern |
| ☒ | ☐ | Plants |

## Methods

| n/a | Involved in the study | |
|---|---|---|
| ☒ | ☐ | ChIP-seq |
| ☒ | ☐ | Flow cytometry |
| ☒ | ☐ | MRI-based neuroimaging |

# Animals and other research organisms

Policy information about studies involving animals; ARRIVE guidelines recommended for reporting animal research, and Sex and Gender in Research

| | |
|---|---|
| Laboratory animals | This study did not envol laboratory animals |
| Wild animals | Yes |
| Reporting on sex | Yes |
| Field-collected samples | The ants were collected in the forests of the Edmund Niles Huyck Preserve, Renssellearville, NY, USA |
| Ethics oversight | Because we work with insects, there are no ethics committees that have to approve anything we do |

Note that full information on the approval of the study protocol must also be provided in the manuscript.
